# Clinical value of INSL3 in the diagnosis and development of diabetic nephropathy

**DOI:** 10.1002/jcla.23898

**Published:** 2021-07-07

**Authors:** Jing Zhu, Xiang Zheng

**Affiliations:** ^1^ Department of Health Management Centre The First Affiliated Hospital of Soochow University Suzhou China

**Keywords:** diabetic nephropathy, diagnosis, glomerular membrane epithelial cells, INSL3

## Abstract

**Background:**

Insulin‐like factor 3 (INSL3) was stated to be an essential regulator in many diseases. This present study aimed to explore the underlying mechanisms of INSL3 in diabetic nephropathy (DN).

**Methods:**

The serum samples were obtained from 121 DN patients, 67 T2DM patients, and 44 healthy controls. Twenty SD rats were used to establish the DN model *in vivo*. Quantitative PCR (qPCR) and Western blot were completed to analyze the INSL3 expression in cells, serum samples, and kidney of the rats. The structure of kidney was analyzed by HE staining. The diagnostic values of INSL3 in DN were determined by receiver operating characteristic (ROC) assay. Then, Spearman's correlation analysis was executed to verify the association between INSL3 and glomerular filtration rate (eGFR). Finally, the proliferation and apoptosis status of transfected cells were analyzed by MTT, flow cytometry, and Hoechst33258 staining assay.

**Results:**

We found that INSL3 expression was up‐regulated in DN patients and SV40‐MES‐13 cells. Furthermore, the correlation analysis elucidated that INSL3 expression was negatively correlated with DN diagnosis golden criterion eGFR. INSL3 knockdown promoted the proliferation rate and inhibited the apoptosis rate of SV40‐MES‐13 cells after high‐glucose treatment. Finally, the INSL3 expression and fast blood glucose were up‐regulated in DN rats.

**Conclusions:**

Collectively, this study demonstrated the clinical significance of INSL3 in diagnosing and developing DN.

## Graphical abstract

We detected that the expression of INSL3 notably increased in both serum sample and high‐glucose‐treated SV40‐MES‐13 cells compared with samples from type 2 diabetic mellitus patients, healthy controls, and normal‐glucose‐treated SV40‐MES‐13. The receiver operating characteristic analysis clarified that INSL3 could discriminate DN patients from T2DM patients and healthy controls with the area under the curves (AUC) of INSL3 of 0.7017 (95% CI = 0.6284~0.7751) and 0.8176 (95% CI = 0.7529~0.8824), respectively. Furthermore, the correlation analysis elucidated that INSL3 expression was negatively correlated with DN diagnosis golden criterion eGFR (*r* = −0.6557, *p* < 0.0001). Finally, experiments in vitro claimed that the proliferation rate of SV40‐MES‐13 cells after high‐glucose treatment was inhibited after INSL3 knockdown; in the meantime, the apoptosis rate significantly increased deeper suggested the potential of INSL3 in DN diagnosis and development. Collectively, this study demonstrated the clinical significance of INSL3 in diagnosing and developing DN.

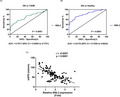

## 1 INTRODUCTION

Diabetic nephropathy (DN) was a common chronic metabolic disease characterized by persistent proteinuria, persistent decrease in glomerular filtration rate, elevated blood pressure, and the occurrence of cardiovascular diseases.^1^, ^2^ DN, as the most severe microangiopathy and the second common complication of diabetes,^3^ afflicts about one‐third of diabetes mellitus (DM) patients.^4^, ^5^ Besides, DN is also one of the leading inducing causes of end‐stage renal disease.^3^ It is fully reported that up to 40% of DM patients worldwide need renal replacement therapy.^6^, ^7^ Studies have shown that a range of mediators or physiological pathways, including hyperglycemia, advanced glycation end products, protein kinases C, oxidative stress, and inflammation, are implicated in the pathogenesis and progression of DN.^8^, ^9^ During the past two decades, many researchers tried to illustrate the pathogenesis of DN; however, the underlying mechanisms of DN still remained unclear.

Recent studies have shown that DN patients have early morphologic changes in abnormal proliferation, hypertrophy, and aging of renal tubular epithelial cells.^10^ The inflammatory response caused by elevated blood sugar is considered an inseparable factor in diabetic complications.^11^ More importantly, the levels of inflammatory cytokines in DN patients were increased, and these levels of inflammatory factors increased steadily with the progression of kidney disease.^12^, ^13^ However, the pathogenesis of DN remains unclear. Hence, the development of novel treatment targets is essential for understanding the progress of DN.

A number of studies have studied the relationship between abnormal expression of different genes and DN progression. For instance, Veiga et al^14^ presented the eccentric expressions of NGAL and SMAD1 genes in blood and urine samples in DN patients by liquid biopsy, suggesting the potentials of NGAL and SMAD1 in DN early diagnosis. Gong et al^15^ claimed that KLF4 serves as a protective regulator in DN by activating podocyte autophagy. Insulin‐like factor 3(INSL3) is a secreted protein encoded by the INSL3 gene in the human body, mainly produced and secreted by testicular interrogating cells.^16^ It plays an important role in the testicular decline and functional maintenance and belongs to the insulin family.^17^ The present study examined the differences in INSL3 expression in serum of DN, type 2 diabetes mellitus (T2DM), and healthy populations. We further compared INSL3 expression with glomerular filtration rate (eGFR) of DN. The importance of INSL3 in DN clinical diagnosis was further verified by ROC analysis. Moreover, we treated the SV40‐MES‐13 cell model with high glucose to mimic DN’s environment in the human body. We observed the regulatory mechanism of INSL3 on abnormal proliferation and apoptosis of glomerular membrane epithelial cells induced by high glucose. Collectively, this study aimed to provide a feasible biomarker for DN diagnosis and a reliable new target for clinical treatment.

## 2 METHODS

### 2.1 Subjects

A total of 232 participants including 121 DN patients, 67 type 2 diabetic mellitus (T2DM) patients, and 44 healthy controls were enrolled from the First Affiliated Hospital of Soochow University between December 2016 and May 2018 (Table 1). After 8 h of fasting, 5~10 mL blood samples were collected. Upon collection, samples were immediately preserved at −80°C until following experiments. This research was approved by the Ethics Committee of the First Affiliated Hospital of Soochow University, in accordance with the Declaration of Helsinki. Written informed consent was signed by each individual who participated in this study.

**TABLE 1:** Clinical features of DN patients, T2DM patients and healthy subjects

Variables	DN (*N* = 121)	T2DM (*N* = 67)	Healthy (*N* = 44)
Gender			
Male	53	28	17
Female	68	39	27
Age	50.6 ± 5.7	52.7 ± 4.1	51.9 ± 5.6
BMI (kg/㎡)	29.5 ± 3.1	27.6 ± 2.2	23.7 ± 4.3
FBG (mg/dL)	172.5 ± 51.3	168.5 ± 44.8	81.7 ± 9.7
PPBG (mg/dL)	268.9 ± 88.1	247.3 ± 74.3	110.7 ± 10.3
Scr (mg/dL)	3.2 ± 1.2	3.3 ± 0.9	0.8 ± 0.7
Disease duration (yr)	9.1 ± 3.2	6.8 ± 4.5	/
Hb1Ac (%)	8.9 ± 3.1	8.6 ± 2.6	4.5 ± 1.3
TG (mmol/L)	4.58 ± 1.19	4.95 ± 1.24	4.25 ± 0.75
eGFR (ml/min)	61.70 ± 24.55	83.51 ± 31.47	129.23 ± 36.84
INSL3 level (fold)	1.915 ± 0.317	1.369 ± 0.276	1.009 ± 0.254
IL−1β (ng/L)	0.69 ± 0.18	0.23 ± 0.21	0.28 ± 0.13
IL−6 (ng/L)	19.84 ± 2.68	10.68±1.615	5.87±3.58
IL−8 (ng/L)	157.65 ± 33.60	120.84 ± 23.84	67.58 ± 31.69
TNF‐α (ng/L)	13.25 ± 1.53	7.56 ± 0.47	4.48 ± 0.36

Abbreviations: BMI, body mass index; DN, diabetic nephropathy; eGRF, glomerular filtration rate; FBG, fasting blood glucose; Healthy, healthy participants; IL, interleukin; INSL3, Insulin‐like protein 3; PPBG, postprandial blood glucose; Scr, serum creatinine; T2DM, type 2 diabetes mellitus; TG, total triglyceridel; TNF‐α, tumor necrosis factor‐α.

### 2.2 Quantitative polymerase chain reaction (qPCR)

According to the manufacturers' protocol, total RNA was extracted from serum samples and cells using TRIzol reagent (Invitrogen, Carlsbad, CA, USA). The reverse transcription reactions were executed using hexamers and M‐MuLV Reverse Transcriptase (New England Biolabs, Ipswich, MA). The cDNA products were diluted ten to a hundredfold and used as PCR templates. The PCR reaction conditions were as follows: 95°C for 90 s; 40 cycles of 95°C for 30 s, 63°C for 30 s, and 72°C for 15 s. GAPDH performed as an internal control to normalize the level of INSL3. The primer sequence of INSL3 was as follows: Forward Primer 5′‐ACCCCAGAGATGCGTGAGAA‐3′, Reverse Primer 5′‐CTCCAGCCACTGTAGCAACTC‐3′. The transcript levels were calculated by the equation of 2^−ΔΔCt^.^18^ All experiments were performed at least in triplicate.

### 2.3 Cell culture and high‐glucose treatment

The glomerular membrane epithelial cell line (SV40‐MES‐13) was purchased from the American Type Culture Collection (ATCC, Rockville, MD, USA). Cells were developed in Dulbecco's Modified Eagle Medium (DMEM; Life Technologies, USA) supplemented with 10% fetal bovine serum (FBS, Gemini Bio‐Products, West Sacramento, CA), 100 U/mL penicillin, and 100 mg/mL streptomycin at 37°C in a humidified incubator with 5% CO_2_. For high‐glucose treatment, cells were infected with normal glucose (5.6 mM glucose) and high glucose (30 nM glucose) and incubated in a humidified atmosphere at 37°C, 5% CO_2,_ until subsequent experiments.

### 2.4 Vector construction and cell transfection

Small interfering RNAs (siRNAs) specifically targeting INSL3 (si‐INSL3) and negative control (si‐NC) were designed and synthesized by GenePharma (Shanghai, China). Cell transfection was executed by Lipofectamine 2000 reagent (Invitrogen, Carlsbad, CA, USA) according to the manufacturer's introduction. The treated cells were processed for further experimentation 24 h after transfection.

### 2.5 MTT assay

Transfected cells were seeded in 96‐well plates (Corning, Corning, NY, USA) at a density of 3.0 × 10^3^ cells per well. A total of 20 µl MTT solution (Sigma‐Aldrich, St. Louis, MO, USA) were added into each well. Cells were then incubated for 4 h at 37°C. Following that, upper‐medium was discarded, and dimethyl sulfoxide (DMSO; Sigma‐Aldrich, St. Louis, MO, USA) was then added to dissolve the formazan crystal in the cell. Lastly, the optical density (OD) value in each well was measured at 490 nm using Multiskan Ascent (Thermo Fisher Scientific, Waltham, MA, USA) at 0, 24, and 48 h to value cell proliferation.

### 2.6 Flow cytometry

The apoptotic cells were detected by flow cytometry. The cells of each group were washed twice with PBS. The cell concentration was adjusted to 1 × 10^5^ cells/ml. 5 μ 1 L annexin V and PI was added and mixed, and incubated in dark for 15 min. The apoptosis rate was detected with flow cytometry (Thermo Fisher Technology Co., Ltd., MA, USA).

### 2.7 Hoechst 33258 staining

To assess nuclear morphology changes during apoptosis, staining using Hoechst 33258 (Beyotime Biotechnology, Shanghai, China) was used as per the instructions. Briefly, transfected cells after high‐glucose treatment were seeded into a 6‐well cell culture plate with sterile coverslips and incubated overnight at 37°C. After culturing in serum‐free media for 24 h, cells on the coverslips were fixed with 4% paraformaldehyde in PBS for 30 min at room temperature, which was stained with Hoechst 33258 for 5 min afterward. Coverslips were mounted with Antifade Mounting Medium (Beyotime Biotechnology, Shanghai, China) and observed under a fluorescent microscope (Nikon, Eclipse 80i, Tokyo, Japan). Apoptotic nuclei were located by morphologic changes such as chromatin condensation and nuclear fragmentation.

### 2.8 Animal experiments

The SD rats were provided by Animal Experiment Middle School of Soochow University. The rats were randomly divided into two groups: Control group and Model group. Ten rats in each group. The rats in Control group were received normal diet, and the rats in Model group were received high‐fat‐sugar diet. The rats in the Model group were intraperitoneally injected with 50 mg/kg Streptozocin (Sigma, MO, USA) after for 4 weeks. Seven days after injection of Streptozocin, the levels of fasting blood glucose (FBG) in the serum of the rats were determined with a kit provided by Nanjing Jiangcheng Bioengineering Institute (Nanjing, China).

#### 2.8.1 Hematoxylin and eosin (HE) staining

The rats were anesthetized by intraperitoneal injection of chloral hydrate and sacrificed. The left kidneys were collected and fixed with 4% paraformaldehyde for 24 h, and paraffin‐embedded sections were made. Then, the sections were stained with hematoxylin for 3 min and eosin for 30 s. The sections were observed and photographed by inverted microscope (Olympus, Tokyo, Japan).

### 2.9 Western blotting assay

Proteins were extricated with lysis buffer and electrophoretically transferred onto polyvinylidene difluoride membranes (PVDF; Millipore). After blocking with 5% skimmed milk for 1 h, the membranes were incubated with primary antibodies against INSL3 and GAPDH (Cell Signaling Technology Inc, Danvers, MA, USA) at 4°C overnight, followed by incubation with HRP‐conjugated secondary antibodies for 2 h at 37°C. The ECL detection system (Millipore, USA) was employed to visualize the signals.

### 2.10 Statistical analysis

All the data were shown using mean ± standard deviation (SD). The results were analyzed using SPSS 22.0 software (SPSS, Chicago, IL, USA) and GraphPad Prism 7.0 (GraphPad Software Inc., La Jolla, CA, USA). The significance of differences was estimated by student's *t* test or one‐way analysis of variance (ANOVA) followed by Tukey's post hoc test. Spearman's correlation analysis measured the association between INSL3 and related factors. Receiver operating characteristic analysis was executed to measure the diagnosis value of INSL3. *p* < 0.05 was considered statistically significant.

## 3 RESULTS

### 3.1 Elevated INSL3 expression in serum samples from DN patients

**FIGURE 1:**
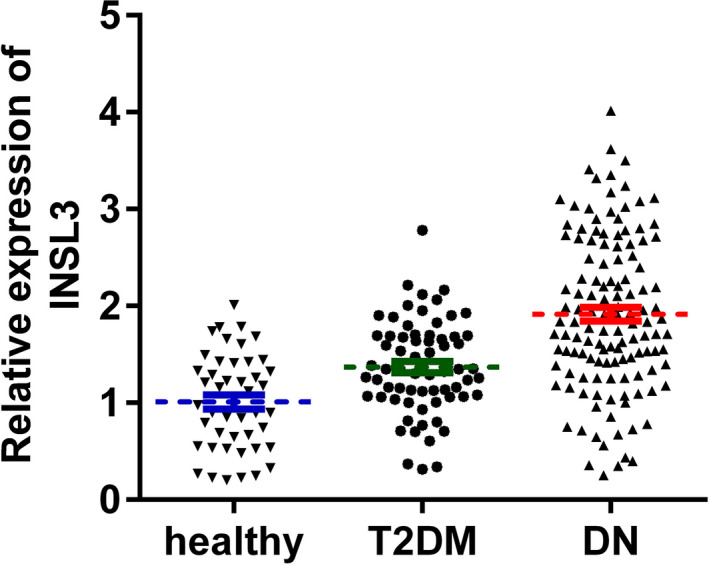
The expression of INSL3 was significantly increased in serum samples from DN compared with those from T2DM patients and healthy controls. *p* < 0.01, DN vs. healthy, DN vs. T2DM. DN, diabetic nephropathy; T2DM, type 2 diabetes mellitus; healthy, healthy controls

We summarized the clinical features, serum indexes, and the expression of INSL3 in all the participants (Table 1). High INSL3 expression was considerably correlated with FBG, PPBG, Scr, serum creatinine, disease duration, Hb1Ac, eGFR, IL‐1β, IL‐6, IL‐8, and TNF‐α. Using qPCR assay, we found that the expression of INSL3 was highly increased in serum samples from DN patients compared to T2DM patients or healthy participants (Figure 1).

### 3.2 The diagnostic value of INSL3 in DN

**FIGURE 2:**
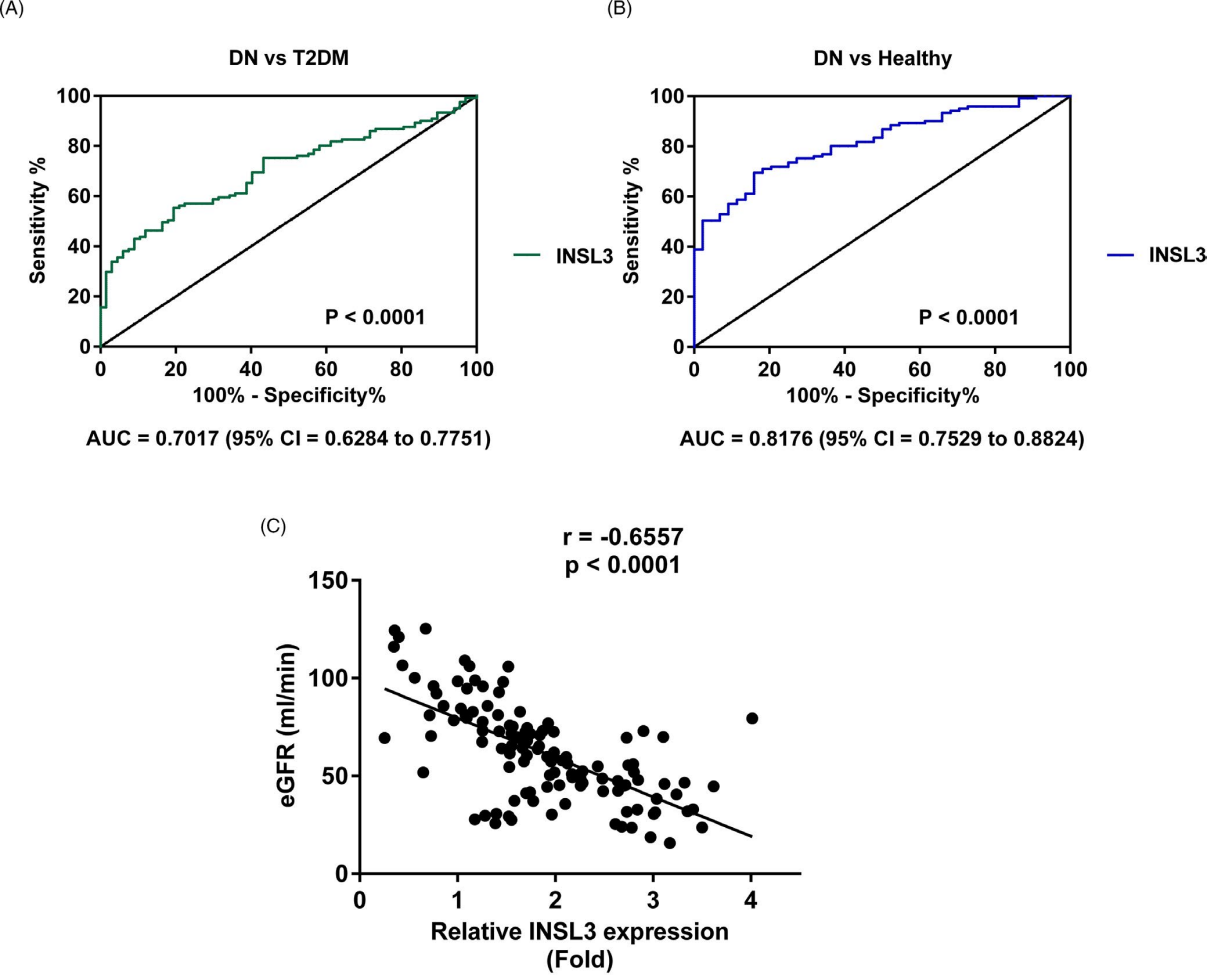
The diagnostic significance of INSL3 in DN. (A) The AUC of INSL3 concerning discriminating DN from T2DM patients. (B) The AUC of INSL3 concerning discriminating DN from healthy controls. (C) The correlation between INSL3 and eGFR. DN, diabetic nephropathy; T2DM, type 2 diabetes mellitus; healthy, healthy controls; eGFR, glomerular filtration rate

Through receiver operating characteristic analysis, we verified the potentials of INSL3 concerning discriminating DN from T2DM patients or healthy controls. The results in Figure 2A,B displayed that the area under curves (AUC) of INSL3 were 0.7017 and 0.8176, respectively. Furthermore, we performed Spearman's correlation analysis to analyze the association between INSL3 and DN diagnosis golden criterion, eGFR. In the results in Figure 2C, we found that INSL3 was negatively correlated with eGFR (*r* = −0.6557; *p* < 0.0001), suggesting the significance of INSL3 in DN diagnosis.

### 3.3 Elevated INSL3 was highly expressed in SV40‐MES‐13 cell after high‐glucose stimulation

**FIGURE 3:**
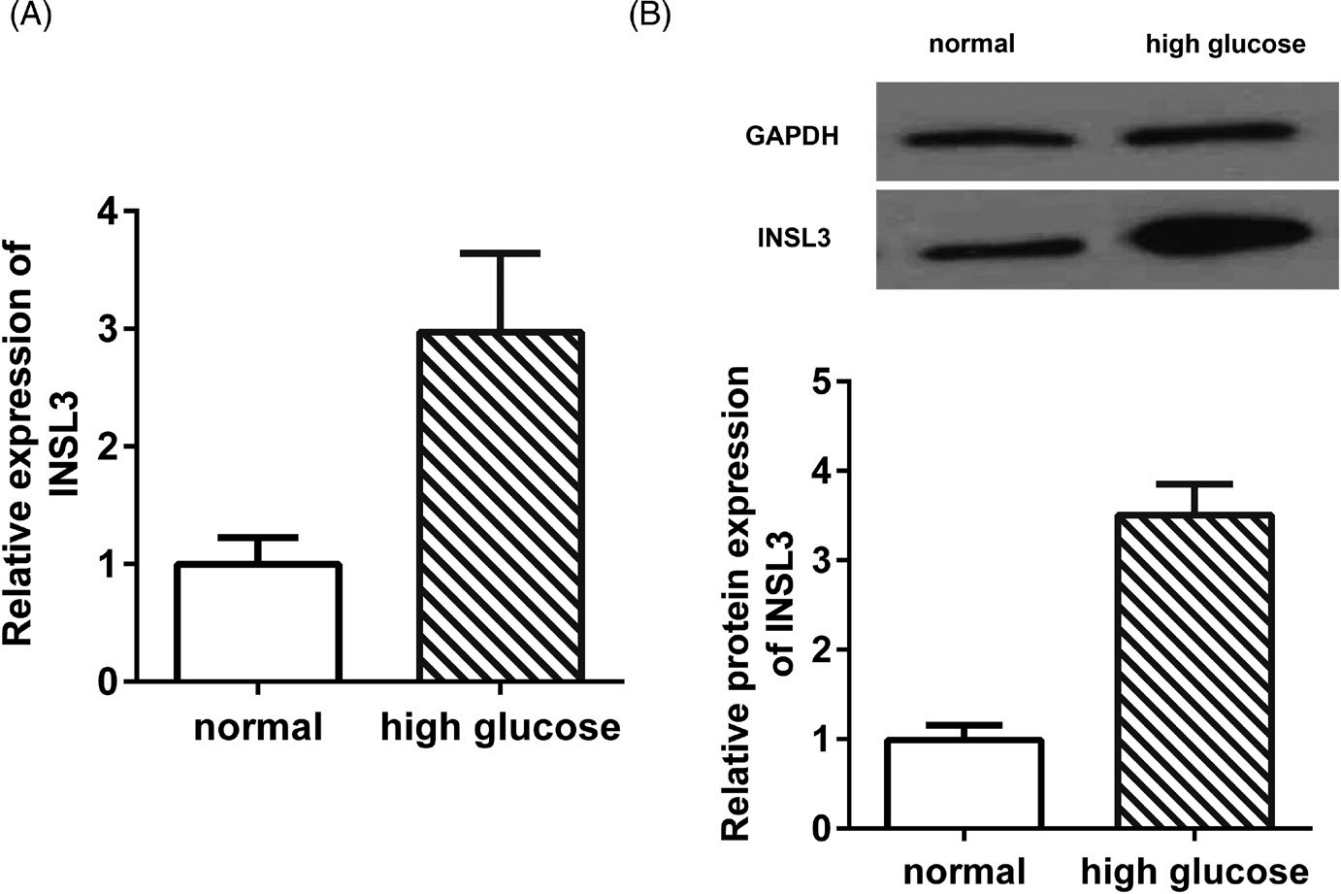
The expression of INSL3 was markedly up‐regulated in SW40‐MES130 cells after high‐glucose stimulation compared with normal glucose. (A) INSL3 mRNA expression in SW40‐MES130 cells under different glucose conditions. (B) INSL3 protein expression in SW40‐MES130 cells under different glucose conditions. *p* < 0.01, high glucose vs. normal. Normal cells were treated with normal glucose

To investigate the role of INSL3 in DN, expression of INSL3 in SV40‐MES‐13 cells under high‐glucose treatment was measured using qPCR and Western blot assay. As shown in Figure 3A–C, INSL3 was up‐regulated in mRNA and protein levels after being treated with high‐glucose compared with normal glucose treatment conditions (^**^ *p* < 0.01).

### 3.4 INSL3 silencing promoted the proliferation while inhibited apoptosis in SW40‐MES130 cells under high‐glucose conditions

**FIGURE 4:**
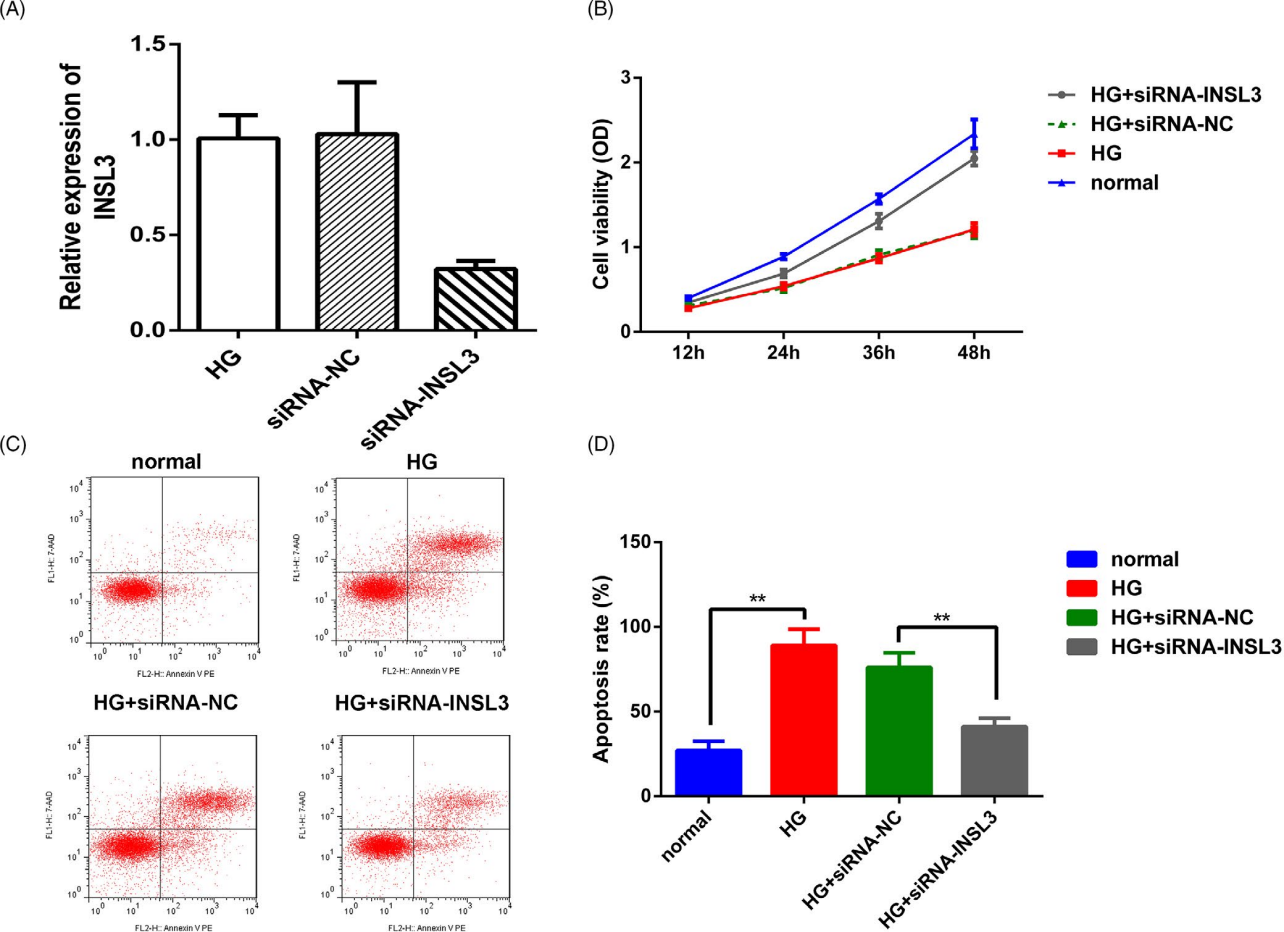
Insulin‐like factor 3 knockdown inhibited SW40‐MES130 cell proliferation under high‐glucose conditions. (A) The transfection efficacy of INSL3 was determined by qPCR assay. *p* < 0.01, siRNA‐INSL3 vs siRNA‐NC or HG group. (B) MTT assay was employed to measure the cell viability in different groups. (C–D) Flow cytometry was employed to measure the cell apoptosis in different groups. (E) Hoechst33258 staining assay. ^**^ *p* < 0.01, ^*^ *p* < 0.05, HG +siRNA‐INSL3 vs HG +siRNA‐NC group, HG vs normal group. HG, high glucose

To inspect the regulatory role of INSL3 in DN, we knocked down INSL3 expression in high‐glucose‐treated SW40‐MES130 cell line by transfection with si‐INSL3, and the knockdown efficiency was confirmed by qPCR (Figure 4A). MTT assay revealed that the viability of SW40‐MES130 cells was noticeably promoted after INSL3 knockdown (Figure 4B). Furthermore, INSL3 knockdown significantly reduced the apoptosis rate of the SW40‐MES130 cell line compared with normal glucose group (Figure 4C–E).

### 3.5 INSL3 expression was up‐regulated in the kidney of the DN rats

**FIGURE 5:**
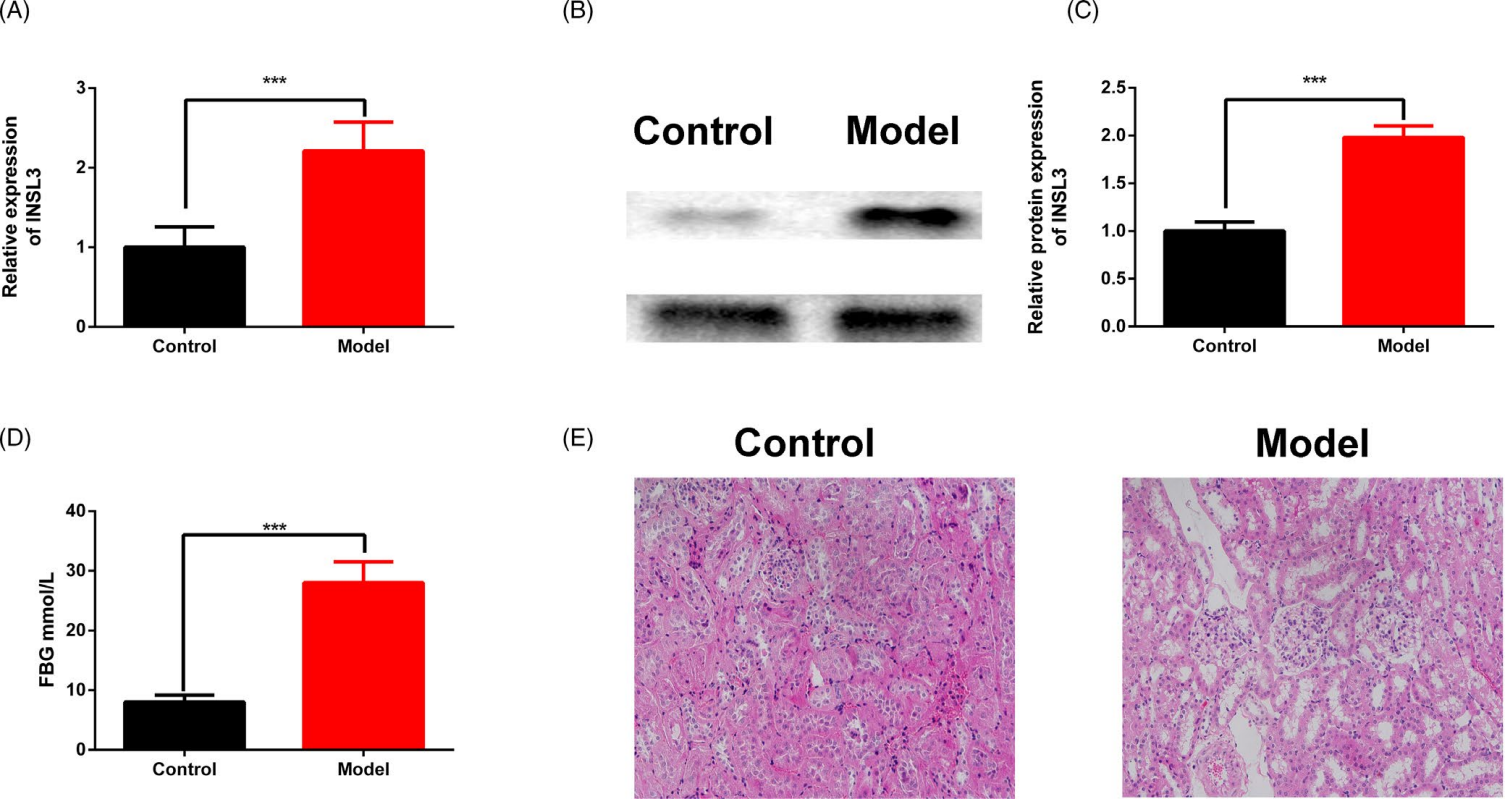
Insulin‐like factor 3 expression was up‐regulated in DN rats. (A) The expression of INSL3 was determined by qPCR assay. (B–C) The INSL3 protein expression was determined by Western blot assay. (D) The FBG levels of the DN rats. (E) HE staining of the DN rats. ^***^ *p* < 0.001, Model group VS Control group

Finally, we further explored the expression of INSL3 in the DN rats. We found that compared with the Control group, the mRNA (Figure 5A) and protein (Figure 5B–C) expressions of INSL3 were significantly up‐regulated in the Model group. And the FBG levels were also significantly increased in the Model group (Figure 5D). Moreover, the HE staining showed that in the Control group, the glomerular epithelial cells were arranged orderly; in the Model group, the volume of glomerulus and extracellular matrix was increased, the mesangial area was widened, the basement membrane was thickened, and the renal interstitium was infiltrated by inflammatory cells (Figure 5E).

## 4 DISCUSSION

Accumulative evidence has shown INSL3 has a vital role in the progression of many diseases, including DN. For instance, INSL3 was proven to be a feasible biomarker for testis status, especially in adult men.^19^ It was claimed that INSL3 is a valuable indicator in women with polycystic ovary syndrome and premature ovarian failure.^20^ As a hormone produced by Leydig cells, INSL3 was also deemed to distinguish Leydig cell hyperplasia and Leydig cell tumor with high accuracy.^21^ In metabolic‐related diseases, INSL3 was found dramatically down‐regulated in obese adolescents.^22^, ^23^ Another study by Ivell et al^20^ demonstrated that INSL3 was also related to kidney function as well. However, the potential mechanism by which INSL3 plays its role in DN remains to be further studied.

In this study, the role of INSL3 in DN was investigated *in vivo* and *in vitro*, and it was discovered that INSL3 was, indeed, up‐regulated in DN serum samples, SV40‐MES‐13 cells after treated with high glucose and kidney of the DN rats. Loss‐of‐function experiments revealed that knockdown of INSL3 promoted cell proliferation while inhibited apoptosis in vitro. These findings imply that INSL3 may act as an anti‐protective role in DN development.

A growing number of studies have studied the potential of INSL3 in clinical diagnosis or prognosis in various diseases, such as atrophy and weakness in skeletal muscle,^24^ klinefelter syndrome,^25^ and thyroid cancer.^26^ The present study illustrated that INSL3 was abundantly expressed in DN tissues and negatively correlated with an advanced INSS stage and unfavorable prognosis. More importantly, the AUC of INSL3 in discriminating DN from healthy controls was 0.8060, suggesting the potential power of INSL3 as a clinical indicator in DN.

However, there were several limitations of this present study. First, DN was a highly heterogeneous tumor; thus, to evaluate the unique roles of INSL3 in different DN subtypes were necessary. Hence, a larger sample sizes of DN patients were required.

In summary, it has been identified that INSL3 expressions were significantly elevated in DN serum samples and cells. Mechanism studies further illustrated the diagnostic value of INSL3 in DN, which may promote further exploration for DN clinical treatment.

## CONFLICT OF INTEREST

None.

## Data Availability

The datasets used and/or analyzed during the current study are available from the corresponding author on reasonable request.
